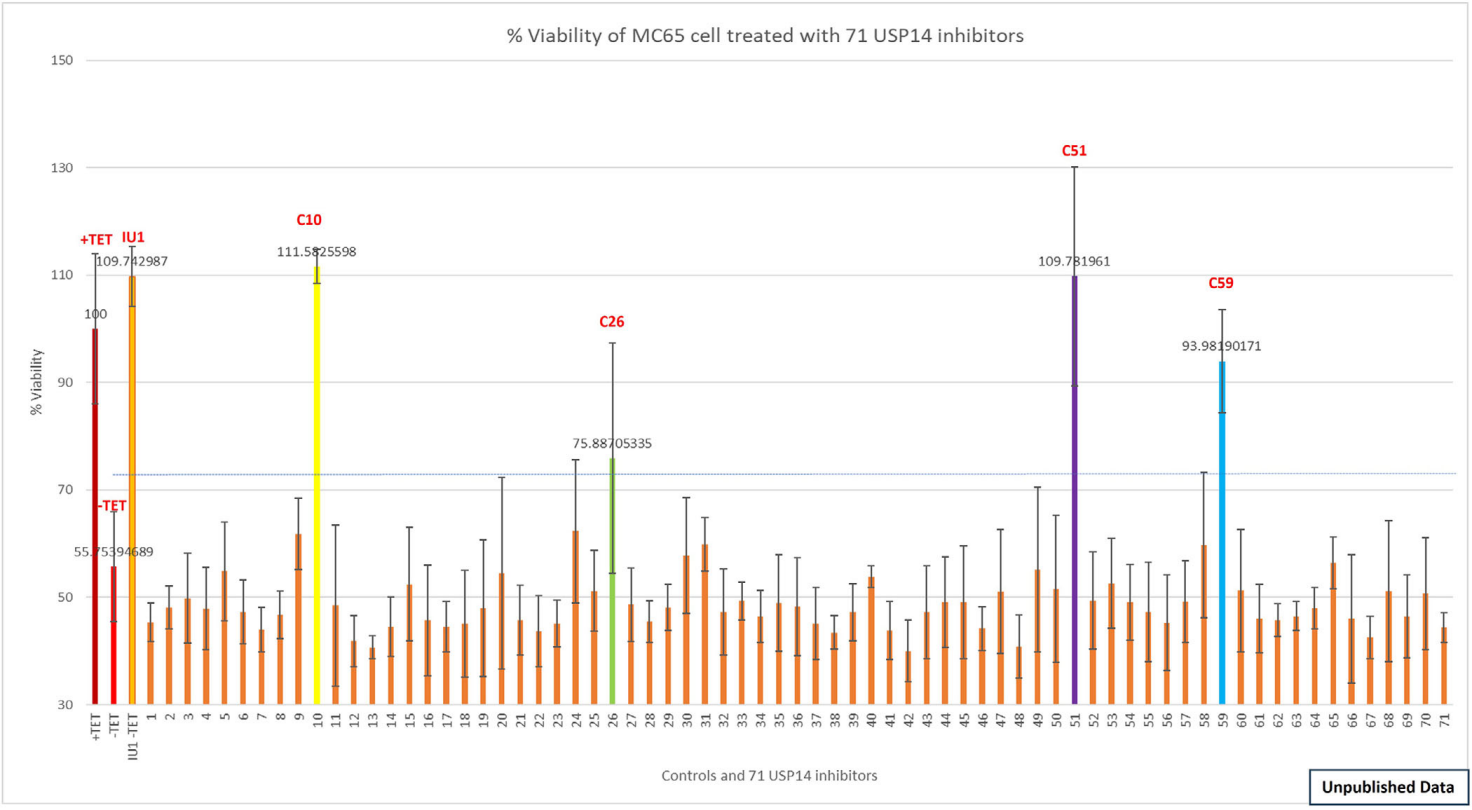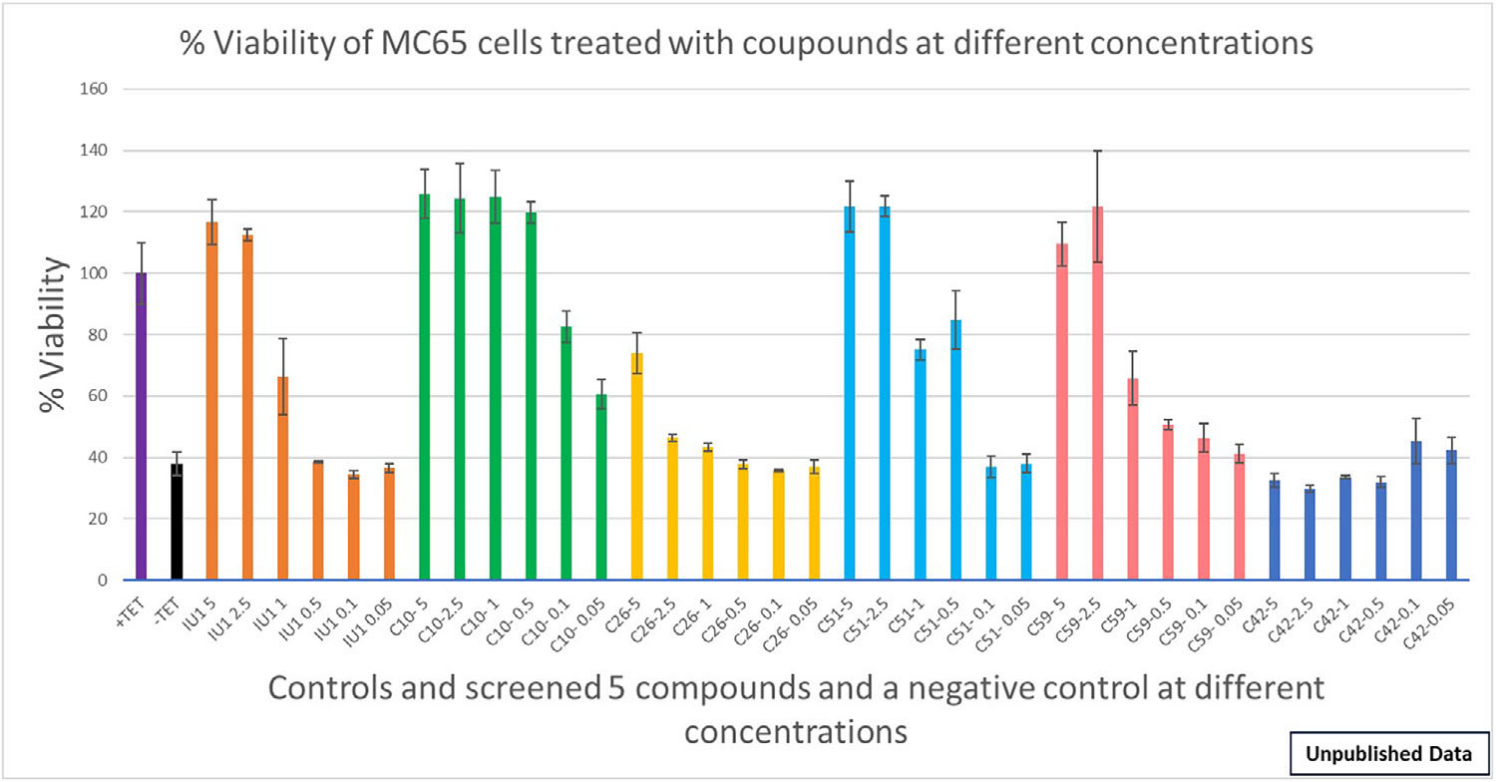# Developing Novel Small Molecule Proteostasis Enhancers for Alzheimer’s Disease (AD)

**DOI:** 10.1002/alz.090110

**Published:** 2025-01-09

**Authors:** Ajish Ariyath, Warnakulasuriya Mary Ann Dipika Binosha Fernando, Ralph N Martins, Prashant Bharadwaj

**Affiliations:** ^1^ Edith Cowan University, Perth, Western Australia Australia; ^2^ Centre of Excellence for Alzheimer’s Disease Research and Care, School of Medical and Health Sciences, Edith Cowan University, Joondalup, Western Australia Australia; ^3^ ECU, Perth, Western Australia Australia

## Abstract

**Background:**

The autophagy lysosomal pathway (ALP) and the ubiquitin‐proteasome system (UPS) are key proteostasis mechanisms in cells, which are dysfunctional in AD and linked to protein aggregation and neuronal death. Autophagy is over activated in Alzheimer’s disease brain whereas UPS is severely impaired. Activating autophagy has received most attention, however recent evidence suggests that UPS can clear aggregate proteins and a potential therapeutic target for AD and protein misfolding diseases.

**Method:**

We previously developed an assay using the MC65 AD cell model and demonstrated that Amyloid Precursor Protein (APP) derived carboxy terminal peptides (APP‐C99) and amyloid‐β protein (Aβ) is rapidly cleared in this model. Using this model we screened a library of small molecule proteostasis modulators and identified IU1, a USP14 inhibitor that improved cell survival and promoted Aβ clearance. Our study investigated whether new analogues of IU1 could provide better neuroprotection in AD. Screening of 71 novel small molecule proteostasis inhibitors using this cell model, discovered two novel lead compounds C10 and C51. These compounds effect on autophagy, proteasome activity, and APP‐C99/Aβ clearance were further analyzed using techniques involving immunofluorescence staining, toxicity analysis, western blot, DUB, proteasome and luminescence assays.

**Result:**

Initial study used an in‐silico artificial intelligence screening platform from Atomwise to design and develop novel small molecule USP14 binding ligands. Cell studies have shown promising results with compounds C10 and C51 from the screened library of proteostasis modulators in alleviating AD proteostasis dysfunction, protein aggregation and regulating autophagy clearance. Cell survival by IU1 was 40% which was improved to 55% using C10 and C51, where it reduced accumulation of APP‐C99 and Aβ. Also, reduced levels of autophagy markers LC3 and p62, restored proteasomal activity in the AD cell model.

**Conclusion:**

In conclusion, ours is the first novel report of using IU1 in an AD model as a USP14 inhibitor. The **novel** IU1 analogues C10 and C51 looks more promising and hold potential as candidates for pre‐clinical validation in AD. The next steps involve testing therapeutic efficacy, target engagement, and brain bioavailability in AD animal models, with completed cell model assessments and upcoming focus on confirming deubiquitinating enzyme (DUB) specificity.